# Capillary Electrophoresis with Interchangeable Cartridges for Versatile and Automated Analyses of Dried Blood Spot Samples

**DOI:** 10.1021/acs.analchem.3c02474

**Published:** 2023-07-28

**Authors:** Miloš Dvořák, Ondrej Moravčík, Pavel Kubáň

**Affiliations:** † Institute of Analytical Chemistry of the Czech Academy of Sciences, Veveří 97, CZ-60200 Brno, Czech Republic; ‡ Faculty of Science, Department of Chemistry, Masaryk University, Kamenice 5, CZ-62500 Brno, Czech Republic

## Abstract

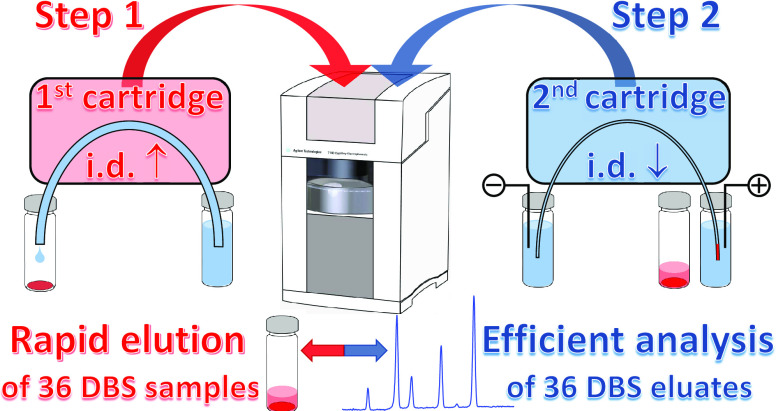

A novel concept for highly versatile automated analyses of dried blood spot (DBS) samples by commercial capillary electrophoresis (CE) is presented. Two interchangeable CE cartridges with different fused-silica capillaries were used for the DBS elutions and the DBS eluate analyses, respectively. The application of one CE cartridge with a wide-bore capillary reduced DBS processing times to a minimum (1–2 min per sample) while fitting the other CE cartridge with a narrow-bore capillary served for highly efficient CE analyses. A comprehensive investigation of major variables affecting liquid handling in CE (capillary length, internal diameter, and temperature) was carried out with the aim of optimizing both procedures and enabling their maximum flexibility. The application of two CE cartridges also enabled the employment of CE detectors with different instrumental set-ups and/or principles as was demonstrated by the optical detection of nonsteroidal anti-inflammatory drugs (NSAIDs) and the conductivity detection of amino acids (AAs). The presented methods were optimized for the automated CE analyses of 36 DBS samples formed by a volumetric collection of 5 μL of capillary blood onto Whatman 903 discs and processed by direct in-vial elution using the CE instrument. The precision of liquid transfers for the automated DBS elutions was better than 0.9% and the precision of CE analyses did not exceed 5.1 and 12.3% for the determination of NSAIDs and AAs, respectively. Both methods were linear (*R*^2^ ≥ 0.996) over the therapeutic (NSAIDs) and the endogenous (AAs) concentration ranges, had limits of quantification below the typical analyte concentrations in human blood, and achieved sample throughputs of more than 6 DBSs per hour.

## Introduction

The collection of dried blood spots (DBSs), i.e., capillary blood from a finger, toe, or heel prick, has become a practical alternative to the collection of venous blood during the recent COVID-19 pandemic.^1,2^ The pandemic has also underlined the major advantages of DBS sampling, which benefits from its minimal invasiveness, suitability for remote patient-centric blood sampling, and simple transport to a laboratory. The DBS sampling does not require trained personnel for blood draws not even the cold chain for the shipment; moreover, many analytes exhibit increased stability in dried samples.^3^ In addition, recent developments of novel blood sampling techniques have also addressed one of the main challenges of DBS sampling, i.e., the simple collection of exact blood volumes independently of blood hematocrit levels.^4−7^

Some of the remaining challenges linked with DBS analyses are the DBS rehydration and pretreatment because the dried materials are processed manually in most assays. They involve punching out the DBS and its transfer to a special container for elution, which are typically followed by centrifugation, extraction, evaporation, and reconstitution of the evaporated extract. The entire process is rather complex, labor- and time-intensive; moreover, the resulting sample must be manually transferred to an analytical instrument.^3^ Although some steps of the DBS processing can be semi-automated (e.g., by using commercial punchers and liquid handling systems), these semi-automated procedures are not suitable for clinical laboratories.^8^

The fact that many procedures have to be carried out manually limits the broader acceptance of DBSs and there has been an urgent quest for the automation of DBS analyses recently.^9−14^ The typical DBS sampling media are paper cards with standardized dimensions/blood collection procedures and the automated DBS analytical systems were designed to accept these DBS collection cards.^15,16^ Before DBS rehydration, elution, and/or extraction, the system applies an internal standard to the DBS and identifies the DBS center, and the system cleans the flow-through cell after the DBS elution. The resulting sample is pumped into the sample loop of an HPLC system for at-line injection, separation, and MS/MS detection of the eluted blood components.^8^

Despite the tremendous achievements in the automation of DBS analyses, there is still a need for easier, cheaper, and more flexible unmanned analytical systems. For example, the flow-through cells elute a subsection of the original DBS only, and although robotically centered, DBSs formed from blood with various hematocrits provide eluates with various blood volumes. Elution efficiencies of these cells are low and are influenced by the blood hematocrit levels. Moreover, analytes are distributed nonhomogeneously within the DBS due to the omnipresent chromatographic effects. These aspects have a direct bearing on the quantitative analysis^17,18^ and the application of correction factor(s) might be necessary.^19,20^ However, the determination of correction factor(s) is not possible because these systems provide only one eluate (i.e., one analysis) per DBS. Moreover, internal conduits, flow-through cells, HPLC columns, and MS/MS interfaces are sensitive to the presence of cellular components, biomolecules, and salts in DBS eluates. These matrix components may poison or clog the analytical system^8^ and are usually eliminated from DBS eluates by using solutions with high or absolute content of organic solvents.^21^ Organic DBS eluates might, however, not be compatible with the reversed-phase columns in HPLC; thereby, the most challenging procedure is finding the proper combination of the solvents for elutions and analyses.^8^ Furthermore, many endogenous analytes are hydrophilic, are not properly eluted with organic solvents, and because aqueous eluates are not suitable for the above reasons, the actual systems will have certain limits for analyses of hydrophilic compounds. Finally, two sophisticated instruments (one for DBS elutions and the other one for DBS analyses) are required, which make these systems bulky and expensive.^3^

Recently, an alternative concept for fully automated DBS analyses was demonstrated in our laboratory.^21,22^ This concept employed an off-the-shelf capillary electrophoresis (CE) instrument for the exhaustive elutions of the whole DBSs, which eliminated the detrimental effects of blood hematocrit and spot nonhomogeneity on quantitative DBS analyses. The DBS elutions were carried out with aqueous and organic solvents for hydrophilic and hydrophobic analytes, respectively, and the eluates were at-line injected into the CE separation capillary for direct DBS analyses. The good tolerance of the CE system to the DBS matrix was evidenced by a superb analytical performance for purely aqueous DBS eluates.^22^ Moreover, the entire analytical protocol, including liquid handling, DBS elution, DBS eluate injection, analytes’ separation, detection, and quantitation was carried out with one compact, commercial, bench-top CE instrument at minimal operational costs.^21^ The possible limitation of this concept was the application of a single CE capillary, which presented a compromise between the DBS elution times and the CE separation efficiencies because wide- and narrow-bore capillaries are beneficial for the former and the latter, respectively.

The all-in-one concept for the automated DBS analyses was thus innovated in this contribution to avoid the above limitation and broaden its application range. The possible discrepancy between the optimal capillary dimensions for DBS elutions and CE separations was eliminated by the employment of two interchangeable CE cartridges, each fitted with an individually optimized capillary. The actual set-up, thus, simultaneously provided rapid processing of 36 DBS samples and efficient CE separations of the resulting 36 DBS eluates while it required only a quick cartridge swap between the DBS processing and CE analyses and a short temperature stabilization of the second cartridge. These procedures added virtually no extra time to DBS analyses whereas they enabled the application of various detectors with specific requirements on capillary dimensions and/or different instrumental arrangements. The high flexibility of the presented setup was demonstrated by the determination of nonsteroidal anti-inflammatory drugs (NSAIDs) and amino acids (AAs) using optical and conductivity detectors, respectively. Moreover, the high versatility of the elution and the analytical processes can extend the applicability of the presented set-up to other CE detectors, broader range of analytes, as well as to various dried material spots in the future.

## Experimental Section

### Chemicals and Solutions

Details are provided in the Supporting Information.

#### Capillary Blood and DBS Samples

Capillary blood samples (merely 5 μL) were collected either by a graduated polypropylene (PP) micropipette tip (Sorenson Bioscience Inc., Salt Lake City, UT, USA; One Touch 1–20 μL) connected to a 0.5–10 μL adjustable micropipette (Eppendorf, Hamburg, Germany) or by a precise end-to-end glass tube (Drummond, Broomall, PA, USA, P/N P1799). The 5 μL blood sample was quantitatively transferred onto a sampling card (Whatman 903 Protein Saver, GE Healthcare Ltd., Cardiff, UK) or onto a 5.5 mm disc prepunched from the sampling card and was air-dried at room temperature for 3 h to form the DBS. The DBS was placed in a zip-lock bag with a desiccant, the bag was closed and was stored at room temperature. For DBS analysis, the 5.5 mm disc (prepunched or punched out after drying) with the whole DBS was placed into a PP sample vial (Agilent Technologies, Waldbronn, Germany, P/N 5182-0567) for a manual or an automated in-vial DBS elution. Details on DBS spiking, calibration measurements, and analyses of ibuprofen-containing DBSs are provided in the Supporting Information.

#### Capillary Electrophoresis Instrumentation and Methods

Analyses of DBS eluates and automated DBS processing/analyses were carried out with a 7100 CE instrument (Agilent Technologies). Two different detectors were employed for the determination of UV absorbing and nonabsorbing analytes; NSAIDs were detected by a UV–vis detector and AAs by a capacitively coupled contactless conductivity detector (C^4^D). The built-in diode-array UV–vis detector was operated at 200 and 226 nm, and the C^4^D (Admet, Prague, Czech Republic) was operated at 1.84 MHz and 50 V_pp_. Commercially available CE cartridges (Agilent Technologies, P/N G7100-60002) were fitted with various fused-silica (FS) capillaries for DBS elutions and CE analyses. Details on BGE solutions, FS capillaries, and CE conditions are provided in the Supporting Information.

### DBS Elution

For the manual DBS elution of NSAIDs, 80 μL of acetonitrile (ACN) was pipetted by a 10–100 μL adjustable micropipette (Eppendorf) directly into the PP vial with the DBS disc. For the manual elution of AAs, the solvent was methanol (MeOH) and its volume was 60 μL. The vial was closed with a polyethylene-olefin (PEO) cap (Agilent Technologies, P/N 5181-1507) and placed on a Vibramax 110 agitator (Heidolph Instruments GmbH, Schwabach, Germany). The vial was agitated at 1200 rpm for 3 min to completely soak the DBS with the organic solvent. Subsequently, the PP vial was uncapped, completed with 20 μL of DI water (NSAIDs), or 40 μL of BGE solution diluted 20-fold with DI water (AAs) using the Eppendorf micropipette and re-capped. Blood constituents from the DBS were eluted by an additional agitation at 1200 rpm for 20 or 60 min, respectively.

For the automated DBS elution, the PP vial with the DBS disc was closed with a PEO cap fitted with a 10 mm PTFE/rubber septum (J.G. Finneran Associates Inc., Vineland, NJ, USA, P/N 604040-10, 1 mm thick). The septum was used to avoid evaporation of volatile eluates; see the Supporting Information. The vial was loaded into the CE autosampler and 80 μL of ACN (for NSAIDs) or 60 μL of MeOH (for AAs) was transferred to the vial through a filling capillary (150 μm i.d. and *L*_tot_ = 50 cm). This was achieved by the application of a pressure of 950 mbar for 11 and 14 s, respectively, at a CE cartridge temperature of 30 °C. The DBS elutions were performed in a sequence (processing 36 DBS samples at once); thus, all DBS discs were fully soaked with the organic solvent before an aqueous solution was added. The aqueous elution solution (20 μL for NSAIDs or 40 μL for AAs) was transferred to the vial by the application of 950 mbar for 7 or 13 s, respectively. The optimum in-vial elution time was 20 and 60 min for NSAIDs and AAs, respectively, and thereafter, the DBS eluates in the vials were homogenized with air flushed through the filling capillary at 950 mbar for 2 s.

The DBS discs were not removed from the vials after the DBS elutions and the eluates were injected directly from the free solutions above the discs.

## Results and Discussion

### Concept of Interchangeable CE Cartridges

The previous concept for the automated DBS processing/analysis employed a single CE cartridge and all analytical tasks were performed with the same FS capillary.^21,22^ The application of a single cartridge might be limiting for various DBS analyses because CE typically requires relatively long (≥50 cm) separation capillaries with rather narrow i.d.s (≤75 μm), which offer good separation efficiencies but necessitate extra time for the DBS processing.^21^ On contrary, short (∼30 cm) and wide (≥100 μm i.d.) capillaries offer quick DBS processing but may suffer from compromised separation efficiencies and require a selective detection method to compensate for the latter.^22^ A rather powerful, yet simple alternative to using a single cartridge can be the application of two interchangeable CE cartridges fitted with FS capillaries individually optimized for the DBS processing and CE analyses (various lengths and i.d.s), respectively, presented in this contribution. This might be particularly important for efficient CE separations of target analytes from matrix components and for detectors, which use narrow-bore separation capillaries (e.g., C^4^D) or unusual CE instrumental arrangement (e.g., MS).

### Sample Vial, DBS Size, and Eluent Volume

The selected vial material (PP), DBS size (5.5 mm disc for 5 μL of capillary blood), and eluent volume (100 μL) were based on our previous publication^22^ and a detailed discussion is presented in the Supporting Information.

### Flow-Through Characteristics for Autonomous DBS Elutions

A comprehensive study of flow-through characteristics was carried out for a range of various FS capillaries. Three most typical DBS elution solvents (DI water, MeOH, and ACN) were employed for these experiments. The flow-through characteristics were evaluated at 10–60 °C. The resulting flow rates through 50 and 30 cm long FS capillaries are summarized in Figures S1 and S2 in the Supporting Information, respectively.

The 50 cm long FS capillaries with i.d. ≤ 75 μm were not suitable for rapid liquid transfers because 100 μL of DI water (i.e., the volume selected in the previous section) was transferred to the PP vial in more than 9 and 6 min at the standard (30 °C) and the maximum (60 °C) cartridge temperature, respectively. The filling times were shortened by a factor of approx. 1.6 by using the shortest possible FS capillary (*L*_tot_ = 30 cm); nevertheless, even at this length, 100 μL of DI water was transferred in more than 4 min through capillaries with i.d.s ≤ 75 μm. On the other hand, the widest capillary (50 cm, 200 μm i.d.) did not offer sufficient resolution for the transfers of the required volumes of DBS elution solvents. The DI water, MeOH, and ACN volumes transferred through this capillary in 1 s (the finest time setting of the CE system) were 9.25, 13.91, and 22.03 μL (at 30 °C), respectively, and the resolution was too rough for a precise filling of PP vials at or below the 100 μL level. As a result, the 50 cm long and 150 μm i.d. FS capillary (referred as 50 cm/150 μm later in the manuscript) offered the most convenient filling times and sufficient volume resolution and was selected for further developments of the automated DBS elution. An extended discussion on the application of various filling capillaries is provided in the Supporting Information.

### Time Requirements for the Cartridge Swap

The employment of two CE cartridges requires an additional manual step, i.e., a cartridge swap between the DBS processing and CE analyses, followed by the temperature stabilization of the second cartridge. A set of 36 DBS samples was processed first (using the first cartridge), and then, the resulting 36 DBS eluates were analyzed by CE (using the second cartridge) by two programable sequences. The actual concept using two interchangeable cartridges evidenced only a negligible increase in the total analysis time (∼30 s due to the cartridge swap between the two sequences) because the temperature of the second cartridge stabilized during the capillary preconditioning of the first CE run. Thus, for the set of 36 DBS samples, the total analysis time increase was less than 1 s per DBS.

### Determination of NSAIDs

The CE-UV determination of selected NSAIDs (ibuprofen, naproxen, ketoprofen, and diclofenac) requires a good separation efficiency because the drugs have similar p*K*_a_ values, migrate in a rather narrow window, and the DBS eluates also contain a range of alike matrix species.^21^ Moreover, the analytes and the co-eluted DBS species absorb in the UV region and might not be differentiated by the nonselective UV–vis detector unless they are fully separated, which is usually achieved using a long, narrow-bore separation capillary. A baseline separation of the drugs and matrix components eluted from a spiked DBS was achieved using an optimized BGE solution and a 50 cm/75 μm separation capillary (see Figure S3 and corresponding text in the Supporting Information). The 50 cm/75 μm capillary is, however, not suitable for rapid DBS elutions (see Figure S1 and corresponding discussion in Flow-Through Characteristics for Autonomous DBS Elutions), and thus, the novel concept of using two interchangeable cartridges was examined for speeding up the automated CE analyses of DBS samples. The first cartridge was fitted with the 50 cm/150 μm filling capillary selected previously for rapid DBS elutions and the second cartridge with the 50 cm/75 μm separation capillary selected above for efficient CE analyses.

### DBS Elution Solvent

**Figure 1:**
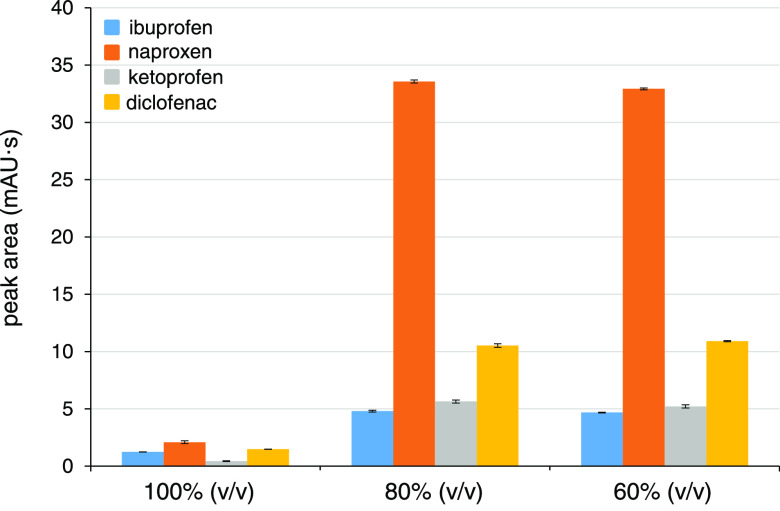
Effect of the ACN content (v/v) in the elution solvent on the NSAIDs elution from DBSs. DBS conditions: 5 μL DBS eluted in 100 μL of various elution solvents (ACN and DI water added consecutively) by agitation at 1200 rpm for 20 min, *n* = 3.

DI water, MeOH, ACN, and their mixtures were examined for the elution of DBS samples spiked with 10 mg/L of the four NSAIDs (their typical therapeutic levels in blood).^23^ The samples were eluted manually (see the Experimental section) and the analyses of DBSs eluted with DI water, 20%, and 40% (v/v) solutions of MeOH and ACN evidenced a detrimental effect of the released blood matrix on CE performance. The blood macromolecular compounds (cellular components, proteins, lipids, etc.) attached to the capillary wall, changed the zeta potential, and shifted the electroosmotic flow peak from its standard migration time (∼2.75 min) to approx. double (∼5 min). The analytes were not detected even after 15 min despite their usual migration times being 3–4 min. The CE system stability improved for injections of DBS eluates in 60% (v/v) MeOH and ACN (slight migration time shifts were still observed), and the CE system was perfectly stable for injections of eluates prepared in 80 and 100% (v/v) of the two solvents. The elution efficiencies for the 60–100% (v/v) ACN and MeOH, expressed as peak areas of the target analytes, are presented in Figures 1 and S4 (in the Supporting Information), respectively.

Best elution efficiencies, CE stability, and CE peak shapes were achieved for the DBS eluates in 80% (v/v) ACN; thus, all subsequent experiments were carried out with DBSs eluted with 80 μL of ACN and 20 μL of DI water (added consecutively). The lower efficiencies for pure solvents were due to their aprotic character (more pronounced for ACN in comparison to MeOH), and the lower CE stability for 60% (v/v) solvents was due to a partial release of the blood matrix into the eluates.

### DBS Elution Time

**Figure 2:**
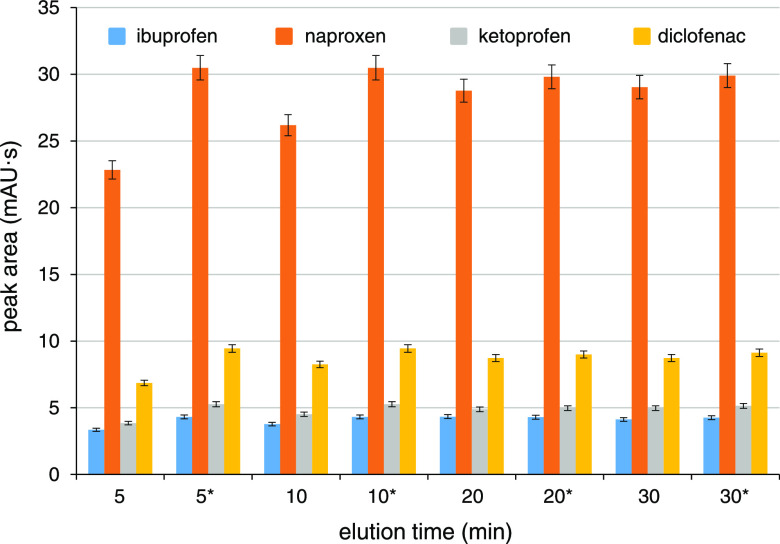
Effect of the in-vial elution time on the NSAIDs elution from DBSs. DBS conditions: 5 μL DBS eluted in 80 μL of ACN and 20 μL of DI water (added consecutively) by the manual in-vial elution and compared with the same DBS eluate additionally agitated at 1200 rpm for 20 min (labeled with *), *n* = 3.

The most convenient DBS elution time was determined by a modified manual elution procedure simulating the real DBS treatment in the CE carousel. First, 80 μL of ACN was pipetted into the vial with the DBS (spiked with 10 mg/L of NSAIDs), the vial was capped with the PEO cap, and placed into a plastic holder (no agitation) for 10 min. The 10 min time enabled a complete saturation of the DBS with ACN and an efficient retention of interfering matrix components in the clotted DBS. Consequently, 20 μL of DI water was pipetted into the vial, which served for an efficient release of the NSAIDs into the DBS eluate. The vial was re-capped and placed into the holder (no agitation) for 5, 10, 15, 20, or 30 min. After the above-specified elution time, the DBS eluate was homogenized by a quick agitation (1200 rpm for 2 s) and injected into the separation capillary for the NSAIDs determination. Immediately after the injection, the vial was placed on the agitator for an additional elution/homogenization (1200 rpm for 20 min) and the resulting DBS eluate was analyzed for comparison. Elution profiles obtained for the in-vial and the comparative DBS elutions are depicted in Figure 2.

The absolute peak areas after the in-vial elution times ≥20 min were stable and comparable with the peak areas after the comparative DBS elution; thus, 20 min was selected for all subsequent in-vial DBS elutions. The 20 min in-vial elution time might be limiting for one-DBS-at-a-time analyses; however, it was not for the automated CE analyses of multiple DBSs as demonstrated in the following section.

### Automated CE-UV Determination of NSAIDs in Multiple DBSs

The capacity of the Agilent 7100 CE system operated with a single cartridge was 36 DBS samples^22^ and could be increased up to 42 samples for the two interchangeable cartridges (details in the Supporting Information and Table S1). However, we have considered the increase not important for this proof-of-concept study and have used the CE sequence and script optimized previously for the analyses of 36 DBS samples.^22^ The distribution of the solutions for the automated elution and determination of NSAIDs in 36 DBS samples is summarized in Table S2 in the Supporting Information.

The automated in-vial DBS elutions were examined with the CE cartridge fitted with the 50 cm/150 μm filling capillary, which enabled convenient, flexible, and rapid transfers of elution solvents to PP vials with DBS samples. The cartridge was kept at 30 °C and the capillary flushing times were set at 11 and 7 s for the transfers of ACN and DI water (see Figure S1), respectively. The transferred volumes (determined gravimetrically) were 80.8 ± 0.92 and 21.0 ± 0.28 μL (*n = 10*), respectively. For the consecutive transfers of ACN and DI water, the total volume in the vial was 101.6 ± 0.91 μL (*n = 36*). Possible evaporation of elution solvents (80% (v/v) ACN, 60% (v/v) MeOH, and DI water) from PP vials was examined (details in the Supporting Information) and demonstrated only a negligible influence of the post-elution time on their volumes (less than 0.4% decrease in 5 h).

The automated DBS analyses were performed by transferring ∼81 μL of ACN to the 36 sample vials (in total 13 min) followed by a capillary flush with DI water (30 s) and subsequent transfer of ∼21 μL of DI water to the 36 vials (in total 10 min) through the first cartridge. After the transfer of the elution solvents, a 15 min WAIT command was executed, which paused the sequence and enabled sufficient elution time for all DBS samples. The capillary was then filled with air (30 s) and was used for a quick (2 s per vial; in total 7 min) homogenization of all 36 DBS eluates by flushing air through the capillary at 950 mbar. Then, the first cartridge was replaced (30 s) with the second cartridge fitted with the 50 cm/75 μm separation capillary. The sequence for CE analyses of all 36 DBS eluates was initiated immediately after the cartridge swap. The temperature inside the second cartridge stabilized at 30 °C during the capillary preconditioning of the first CE run and the 36 DBS eluates were analyzed within 249 min. The total processing and analysis times were 77 and 415 s per DBS, respectively, resulting in a sample throughput of more than 7 DBSs per hour, which conforms with the requirements of the contemporary clinical laboratory assays.^11^

### Analytical Performance

**Figure 3:**
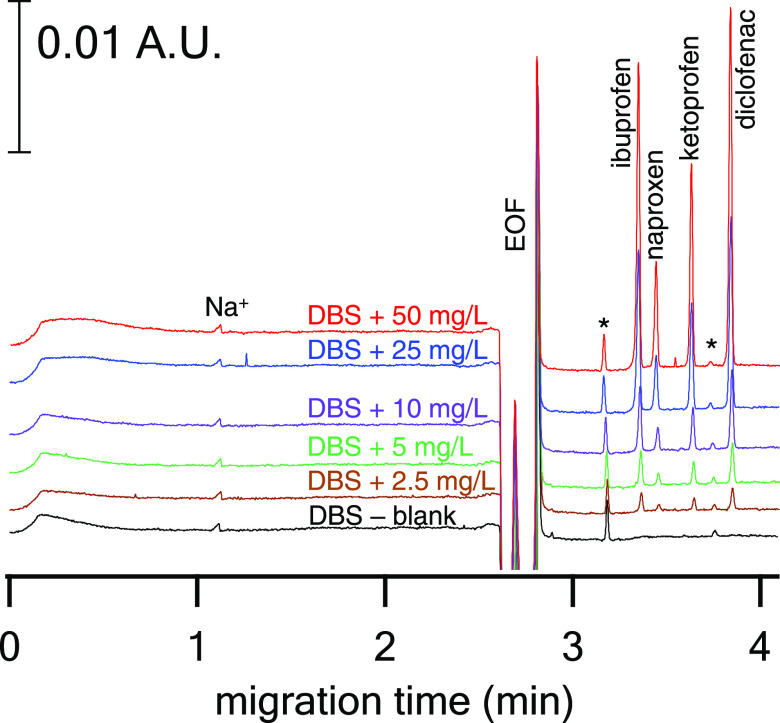
Electropherograms for the CE-UV determination of NSAIDs in DBSs. CE conditions: FS capillary, 75 μm i.d., *L*_tot_ = 50 cm, *L*_eff_ = 41 cm; BGE solution, 30 mM sodium acetate, 60 mM acetic acid, and 20% (v/v) ACN, apparent pH 4.8; separation voltage, +27.5 kV; cartridge temperature, 30 °C; injection, 50 mbar for 7 s; detection wavelength, 200 nm. DBS conditions: 5 μL DBS eluted in ∼81 μL of ACN and ∼21 μL of DI water (added consecutively) by the CE in-vial elution procedure. EOF – electroosmotic flow, * – unknown matrix compounds.

Electropherograms of drug-free DBS samples spiked at various NSAIDs concentrations are depicted in Figure 3.

Details on the analytical parameters of automated NSAIDs determination are provided in Table S3 in the Supporting Information and a summary is presented here. The method repeatability was determined with one DBS spiked at 10 mg/L of NSAIDs and analyzed ten times (*n = 10*), and with four different DBSs collected from the same finger prick 75 min after Ibalgin tablet ingestion (*t*_max_, see Figure S5 in the Supporting Information) and analyzed three times per spot (*n = 12*). The RSD values for the peak areas and migration times were 1.6–2.7 and 0.4–0.5% for the one DBS and 3.6 and 0.6% for the four DBSs, respectively. The RSD values for DBSs spiked with NSAIDs at concentrations near the limit of quantification (LOQ) and at low (5 mg/L), medium (25 mg/L), and high (50 mg/L) calibration levels were determined for 3 DBSs (3 replicate analyses per DBS; *n = 9*) and were below 5.1 and 0.5% for peak areas and migration times, respectively. Inter-day RSD values were determined at a spiked concentration of 10 mg/L of NSAIDs (3 replicate analyses per DBS on three different days; *n = 9*) and were below 4.1 and 0.9%, respectively. The linearity of the analytical method was determined for DBSs collected from four different individuals and spiked with the four NSAIDs. The resulting calibration curves (5-point calibration at 2.5, 5, 10, 25, and 50 mg/L) were linear with coefficients of determination above 0.998. The concentrations of the 5 calibrators were back-calculated from the calibration curves, did not differ from the nominal values by more than 4.9%, and showed that the linear model can be accepted.^24,25^ The RSD values of the calibration curves’ slopes were less than 0.8% (*n = 4*) and all slopes were within the limit for bioanalytical quantitation (average slope ± 2SD),^26^ suggesting that universal calibrations can be used for the quantitative determination of NSAIDs. Limits of detection (LODs) and LOQs were defined as the lowest analyte concentrations giving analytical signals three- and ten-times higher than baseline noise (S/N = 3 and S/N = 10), respectively. The LOD and LOQ values of NSAIDs were 8–30 and 26–100 μg/L in DBS eluates, respectively, which translates to 0.16–0.6 and 0.52–2.0 mg/L in the original capillary blood and were below the therapeutic ranges for NSAIDs in clinical blood samples.^23^ The proposed concept might, thus, be applied to, e.g., rapid and automated therapeutic drug monitoring (TDM). The long-term DBS stability was examined by the analysis of DBSs collected from the same finger prick 75 min after the ingestion of the Ibalgin tablet. Ibuprofen concentrations in the DBSs were determined 1 day, and 2, 3, and 4 weeks after the DBS collection while the DBSs were stored in a closed zip-lock bag with a desiccant at laboratory temperature. The determined concentrations differed by less than 4.7% and demonstrated excellent stability of ibuprofen in DBSs for at least 4 weeks. The suitability of this set-up for TDM was further demonstrated by the determination of the ibuprofen pharmacokinetic curve that is presented in the Supporting Information (Figures S5 and S6).

### Determination of AAs

A broad range of AAs can be determined by CE-C^4^D in strongly acidic BGE solutions with no need for sample derivatization.^27^ To minimize electric currents and Joule heating in the CE-C^4^D system, narrow-bore separation capillaries (≤25 μm i.d.) are usually employed.^27^ Moreover, the use of the narrow-bore capillaries improves the CE separation efficiency, which is crucial for the determination of AAs due to their rather similar p*K*_a_ values and migration times. In the actual experiments, the flexibility of the automated DBS analyses using two interchangeable cartridges was further demonstrated by the CE-C^4^D determination of a set of rapidly migrating AAs (choline, creatinine, β-alanine, ornithine, lysine, histidine, and arginine). The first cartridge was fitted with the previously optimized filling capillary (50 cm/150 μm) and the second cartridge with a 50 cm/25 μm separation capillary typically used for CE-C^4^D of AAs.^27^ A baseline separation of the selected AAs and matrix inorganic cations in a DBS eluate is demonstrated in Figure S7 in the Supporting Information along with the details on the BGE solution.

### DBS Elution Solvent

**Figure 4:**
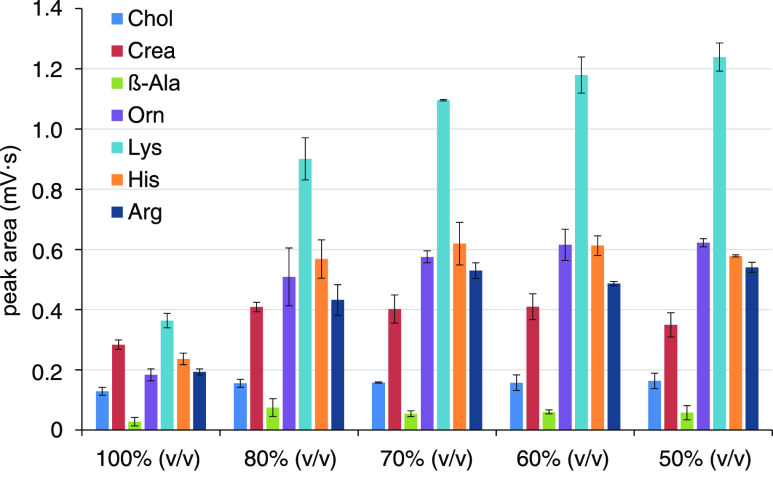
Effect of the MeOH content (v/v) in the elution solvent on the elution of target AAs from DBSs. DBS conditions: 5 μL DBS eluted in 100 μL of various elution solvents (MeOH and DI water added consecutively) by agitation at 1200 rpm for 60 min, *n* = 3.

DI water, MeOH, ACN, and their mixtures were employed for the manual elutions (see the Experimental section) of endogenous AAs from DBSs. DBS eluates prepared in DI water and DI water/organic mixtures with less than 50% (v/v) MeOH and 60% (v/v) ACN had a detrimental effect on CE separations of AAs and these elution solvents were, thus, excluded from further experiments. The CE stability improved for DBS eluates prepared in mixtures containing higher content of organic solvents. However, a slight shift in migration times was still observed for multiple CE injections of DBS eluates containing 60% (v/v) ACN, no AAs were eluted with 100% (v/v) ACN, and a matrix peak partially comigrating with choline was detected in all ACN eluates; ACN was therefore not used in further experiments. The elution efficiencies of the selected AAs and two major inorganic cations (potassium and sodium), expressed as peak areas for various MeOH solutions, are presented in Figures 4 and S8 (in the Supporting Information), respectively. The best elution efficiencies were achieved for DBSs eluted with 50 and 60% (v/v) MeOH and the latter ensured narrower peak shapes of the subsequent CE-C^4^D analyses due to an improved sample stacking. All subsequent experiments were, thus, carried out with DBSs eluted with 60% (v/v) MeOH solutions.

### DBS Elution Time

**Figure 5:**
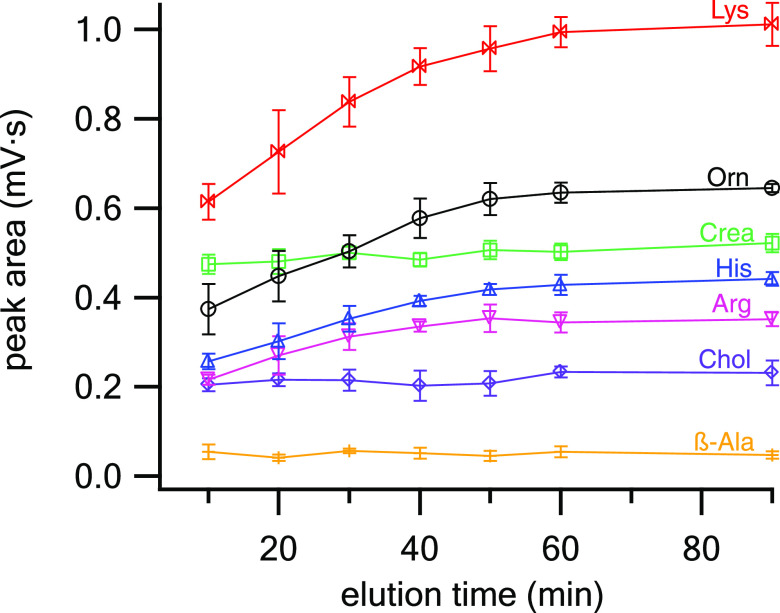
Effect of the in-vial elution time on the AAs elution from DBSs. DBS conditions: 5 μL DBS eluted in 60 μL of MeOH and 40 μL of 20-fold diluted BGE solution (added consecutively) by the manual in-vial elution (10–60 min) and compared with the same DBS eluate additionally agitated at 1200 rpm for 30 min, *n* = 3.

The optimum time for the in-vial elution of AAs from DBS samples was determined by a manual procedure, which simulated the real process inside the CE instrument. The collected DBSs (from one finger prick) were inserted into PP vials and eluted by consecutive additions of MeOH and DI water. First, 60 μL of MeOH was pipetted to the vial, the vial was capped with the PEO cap, and was placed into a plastic holder for 10 min. Second, 40 μL of DI water was pipetted to the MeOH eluate, the vial was capped, and placed into the holder for 10 min. Third, the DBS eluate was homogenized by a quick agitation (1200 rpm for 2 s) and immediately analyzed for the AAs concentrations. The same vial was then used for subsequent CE injections at 20, 30, 40, 50, and 60 min (the vial was kept in the CE carousel and agitated at 1200 rpm for 2 s before each injection). Fourth, an additional elution/homogenization (1200 rpm for 30 min) was applied to this vial and the resulting eluate was used for a comparative CE analysis. The elution efficiencies of the in-vial processed DBSs were stable for elution times ≥30 min for choline, creatinine, and ß-alanine. Basic AAs, i.e., ornithine, lysine, arginine, and histidine, gradually eluted for up to 90 min. To reduce the elution time, the aqueous solvent was modified and elution characteristics improved by using a 20-fold diluted (with DI water) BGE solution. The elution efficiencies of the in-vial processed DBSs were stable for elution times ≥60 min for all analytes and are depicted in Figure 5. The absolute peak areas after the additional 30 min agitation time were constant for all AAs and an elution time of 60 min was, thus, applied to all subsequent automated in-vial DBS elutions.

### Automated CE-C^4^D Determination of AAs in Multiple DBSs

The in-vial DBS elutions were carried out with the first cartridge. The cartridge temperature was 30 °C and capillary flushing times were 14 and 13 s for the transfers of MeOH and 20-fold diluted BGE solution, respectively. The transferred volumes were 62.3 ± 0.75 μL of MeOH and 39.1 ± 0.47 μL of the diluted BGE solution (*n = 10*). For the consecutive transfers of the two solvents, the final volume was 100.9 ± 0.71 μL (*n = 36*).

The distribution of the solutions for the automated elution and determination of AAs in 36 DBS samples is summarized in Table S4 in the Supporting Information. The DBSs were eluted by transferring ∼62 μL of MeOH to the 36 sample vials (in total 15 min), flushing the capillary with the diluted BGE solution (30 s), and transferring ∼39 μL of the diluted BGE solution to the 36 vials (in total 14 min). The sequence was paused for 45 min (WAIT command), the capillary was filled with air (30 s), and the eluates were homogenized by air (2 s per vial, in total 7 min). The first cartridge was replaced with the second cartridge (∼30 s) and the CE analytical sequence was initiated immediately. The temperature (30 °C) inside the second cartridge stabilized during the capillary preconditioning of the first CE run and eliminated any possible signal fluctuations of the C^4^D, which is more sensitive to temperature changes than the UV–vis detector. The total processing and analysis times were 137 and 430 s per DBS, respectively, resulting in a sample throughput of more than 6 DBSs per hour.

### Analytical Parameters

**Figure 6:**
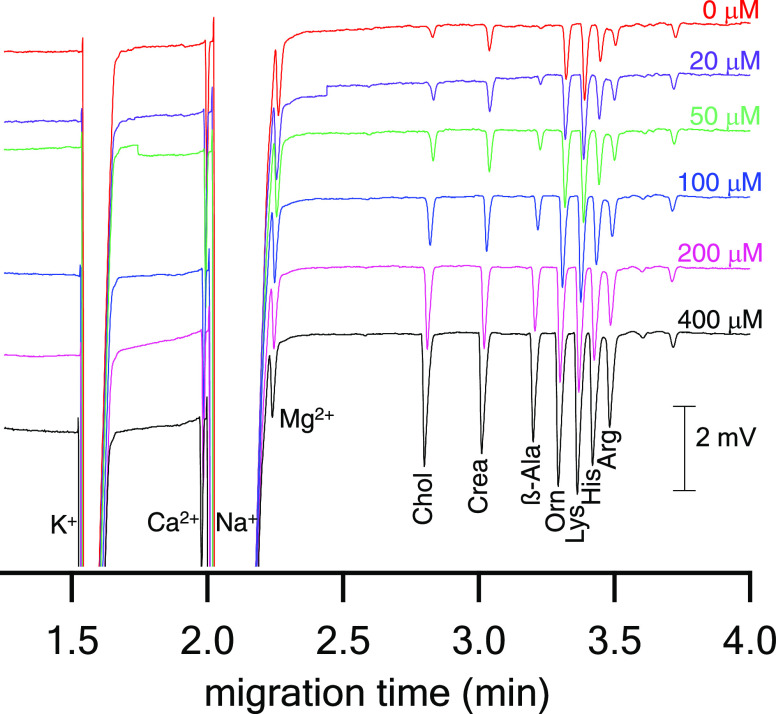
Electropherograms for the CE-C^4^D determination of inorganic cations and AAs in DBSs. CE conditions: FS capillary, 25 μm i.d., *L*_tot_ = 50 cm, *L*_eff_ = 35 cm; BGE solution, 0.4 M acetic acid and 0.1% (v/v) Tween 20, pH 2.6; separation voltage, +25 kV; cartridge temperature, 30 °C; injection, 100 mbar for 10 s. DBS conditions: 5 μL DBS eluted in ∼62 μL of MeOH and ∼39 μL of 20-fold diluted BGE (added consecutively) by the CE in-vial elution procedure.

Endogenous and spiked concentrations of AAs were used for the determination of the method’s repeatability. The analytical parameters of their automated determination are summarized here (details are provided in Table S5 in the Supporting Information), and electropherograms of a neat and spiked DBS samples are depicted in Figure 6.

Intra-day RSD values for the endogenous AAs concentrations were determined for peak areas and migration times in 18 DBSs from one finger-prick and were better than 8.7 and 0.9% (*n = 18*), respectively. ß-Alanine concentration was below its LOQ and fulfilled the precision criteria (RSD ≤ 20%) admitted for the LOQ level.^24,25^ RSD values for DBSs spiked with AAs at various concentration levels were determined for 3 DBSs (3 replicate analyses per DBS; *n = 9*) and were all below 12.3 and 0.7%, respectively. Inter-day RSD values (3 replicate analyses per DBS on three different days; *n = 9*) were below 12.3 and 2.0%, respectively. Linearity of the analytical method was determined for neat and spiked DBSs (5-point calibration at 20, 50, 100, 200, and 400 μM of AAs) and the resulting calibration curves were linear with coefficients of determination ≥0.999 (0.996 for ornithine). The concentrations of the 5 calibrators were back-calculated from the calibration curves, did not differ from the nominal values by more than 12%, and showed that the linear model can be accepted.^24,25^ LODs and LOQs were 4–6 and 13.3–20 μM for the original capillary blood, respectively, and demonstrated sufficient sensitivity of the method for the determination of AAs in blood samples.^28^ The long-term stability of AAs in DBSs was examined based on the same protocol as for NSAIDs. The endogenous AAs concentrations after 4 weeks storage differed by less than 11%, demonstrating their sufficient stability in DBSs. The only exception was arginine with a decrease of 12, 23, and 34% after two, three, and four weeks, respectively.

## Conclusions

The employment of two interchangeable CE cartridges for rapid autonomous DBS processing and efficient CE separation of resulting DBS eluates is proposed in this contribution. The two cartridges use individually optimized FS capillaries, ensure excellent flexibility of both procedures, and significantly broaden the application range of the automated DBS analyses by CE. The presented concept requires only a quick manual cartridge swap between the sequences for DBS processing and CE analyses and is followed by a short temperature stabilization of the second cartridge, which is achieved during the first CE run. The cartridge swap has only a negligible effect on the total analysis time (it adds less than 1 s per DBS sample in a sequence of 36 DBS samples), while it enables rapid solvent transfers for DBS processing, high efficiency for CE separations of complex DBS eluates, and the application of various detectors with specific requirements on separation capillary dimensions and/or with specific instrumental set-up. An extension to, for example, mass spectrometry is thus envisioned in the future, which will additionally improve the selectivity, specificity, and sensitivity of the automated CE-DBS analyses. The actual concept might therefore play an important role in clinical, toxicological, and forensic analyses because it offers rapid DBS processing times, excellent separation efficiencies, great variability in CE detection modes, and high sample throughputs. A further increase in sample throughput might additionally be achieved by the application of commercial CE instruments with high-pressure units, high-capacity sample trays (with temperature control), and/or multiple capillary array options.

## Supporting Information Available

Chemicals, solutions, DBS samples, DBS spiking, informed consent, and CE methods; flow rates in various capillaries; CE-UV of NSAIDs; eluate evaporation; maximum carousel capacity; ibuprofen pharmacokinetics; and analytical parameters (PDF)
