# Supplementary material for: Capillary Electrophoresis with Interchangeable Cartridges for Versatile and Automated Analyses of Dried Blood Spot Samples
Source: Anal Chem. 2023 Jul 28;95(31):11823–30. doi: 10.1021/acs.analchem.3c02474 (PMC10413327; doi:10.1021/acs.analchem.3c02474)
Supplement: Supplementary file 1 — ac3c02474_si_001.pdf [file ac3c02474_si_001.pdf]

Supporting Information for the manuscript

Capillary Electrophoresis with Interchangeable Cartridges for Versatile and Automated Analyses of Dried Blood Spot Samples

Miloš Dvořák[a§], Ondrej Moravčík[ab§], Pavel Kubáň[a§*]

[a]*Institute of Analytical Chemistry of the Czech Academy of Sciences, Veveří 97, CZ-60200, Brno, Czech Republic*

[b]*Faculty of Science, Department of Chemistry, Masaryk University, Kamenice 5, CZ-62500, Brno, Czech Republic*

§M.D, O.M., and P.K. contributed equally to this work.

*Tel.: +420 532290140. Fax: +420 541212113. E-mail: kuban@iach.cz

TABLE OF CONTENTS

EXPERIMENTAL…………………………………………………………………….. S-3

Chemicals and Solutions……………………………………………………….... S-4
Capillary Blood and DBS Samples……………………………………………… S-4
DBS Spiking, Calibration Measurements, and DBS Samples with Ibuprofen……………S-4
Capillary Electrophoresis Instrumentation and Methods………………………………… S-5

RESULTS AND DISCUSION…………………………………………………………S-6

Sample Vial, DBS Size, and Eluent Volume…………………………………….. S-6
DBS Elution Solvent…………………………………………………………….... S-6
Flow-Through Characteristics of Various FS Capillaries for Autonomous DBS Elutions.S-7
Figure S1…………………………………………………………………………… S-8
Figure S2…………………………………………………………………………… S-9
BGE Solution Optimization for CE-UV Determination of NSAIDs…………………….. S-10
Figure S3…………………………………………………………………………… S-11
Figure S4…………………………………………………………………………… S-12
Evaporation of Elution Solvents……………………………………………………. S-12
Maximum Sample Capacity of the CE Carousel …………………………………S-13
Table S1……………………………………………………………………………...S-14
Table S2……………………………………………………………………………...S-14
Ibuprofen Pharmacokinetic Curve………………………………………………...S-15
Figure S5…………………………………………………………………………… S-16
Figure S6…………………………………………………………………………… S-17
Table S3……………………………………………………………………………...S-18
Figure S7…………………………………………………………………………… S-19
Figure S8…………………………………………………………………………… S-20
Table S4…………………………………………………………………………….. S-20
Table S5…………………………………………………………………………….. S-21

REFERENCES………………………………………………………………………S-22

EXPERIMENTAL

Chemicals and Solutions

All chemicals were of analytical reagent grade. DI water with a resistivity of at least 18 MΩ·cm was prepared by exchange of ions in the G7749 (Miele, Gütersloh, Germany) water purification system. Acetonitrile (ACN) was purchased from Penta (Prague, Czech Republic), and methanol (MeOH) from Lach-Ner (Neratovice, Czech Republic). The stock solution of 1 M NaOH was prepared by dissolving NaOH pellets (Lach-Ner) in DI water. Stock solutions of non-steroidal anti-inflammatory drugs (NSAIDs) were prepared by dissolving 10 mg of ibuprofen, naproxen, ketoprofen, and 10.78 mg sodium diclofenac (Sigma, Steinheim, Germany) in 1 mL of MeOH and were stored in a deep-freezer at − 20 °C. Standard solutions for CE analyses of the drugs were prepared by diluting the appropriate stock solutions with ACN and DI water. Stock solutions of amino acids (10 mM, Sigma and Fluka, Buchs, Switzerland) were prepared from L-forms of the following chemicals (creatinine (Crea), lysine (Lys), arginine (Arg), histidine (His), β-alanine (β-Ala), and ornithine (Orn)) and from choline chloride (Chol)). Standard solutions for CE analyses of amino acids were prepared by diluting the appropriate stock solutions with MeOH and DI water. Stock solutions of 1 M acetic acid and sodium acetate were prepared from the corresponding chemicals (both Fluka) in DI water. Background electrolyte (BGE) solution for the determination of NSAIDs was prepared by mixing appropriate volumes of stock solutions of sodium acetate and acetic acid in ACN and DI water. BGE solution for the determination of amino acids was prepared by diluting the acetic acid stock solution with DI water and adding Tween 20 (Sigma). All stock solutions were stored refrigerated at 4 °C for 3 months. Standard and BGE solutions were prepared daily.

Capillary Blood and DBS Samples

Minute volumes of capillary blood were self-collected by volunteers from the Institute of Analytical Chemistry. Written informed consent was signed by all volunteers and the study was aprooved by the director and the council of leading scientists of the Institute of Analytical Chemistry. A detailed capillary blood collection procedure was described in the previous publication[1] and involved the following five steps. (1) Disinfection of a fingertip, (2) pricking the fingertip, (3) wiping off the first blood drop, (4) collection of an exact blood volume from the next drop, and (5) disinfection of the finger-prick.

DBS Spiking, Calibration Measurements, and DBS Samples with Ibuprofen

Drug-free DBSs were used as blank samples for the determination of NSAIDs, and DBSs collected after oral administration of an Ibalgin tablet (Sanofi-Aventis, Prague, Czech Republic, containing 400 mg of ibuprofen) as real samples for the determination of ibuprofen therapeutic concentrations. For quantitative analyses, the DBSs were spiked by pipetting 5 μL of a standard solution with various concentrations of NSAIDs (2.5 – 50 mg/L) onto pre-punched 5.5 mm discs with the DBSs. The discs were air-dried at room temperature for 3 h to enable a proper interaction of the standard solution and the DBS matrix.[2] Neat DBS samples were used for the determination of endogenous AAs concentrations. For validation and calibration measurements, the DBSs were spiked with 20 – 400 μM of AAs according to the above-described procedure.

Capillary Electrophoresis Instrumentation and Methods

The BGE solution for the determination of NSAIDs consisted of 30 mM sodium acetate, 60 mM acetic acid, and 20% (v/v) ACN and had an apparent pH of 4.8. CE separations of NSAIDs were performed in a fused silica (FS) capillary (75 μm i.d./375 μm o.d., L_{tot} = 50 cm, and L_{eff} =

41 cm), supplied by Polymicro Technologies (Phoenix, AZ, USA). The separation voltage applied to the inlet electrode was + 27.5 kV, the temperature inside the CE cartridge was 30 °C, and samples were injected at 50 mbar for 7 s. Ibuprofen, ketoprofen and diclofenac were detected at 200 nm and naproxen at 226 nm. The BGE solution for the determination of amino acids consisted of 0.4 M acetic acid and 0.1% (v/v) Tween 20 and had a pH of 2.6. CE separations of amino acids were performed in an FS capillary (25 μm i.d./375 μm o.d., L_{tot} = 50 cm and L_{eff} = 35 cm), supplied by Polymicro Technologies. The separation voltage applied to the inlet electrode was + 25 kV, the temperature inside the CE cartridge was 30 °C, and samples were injected at 100 mbar for 10 s. Short platinum electrodes (Agilent Technologies, P/N G7100-60033) were used at the inlet and outlet of the CE instrument, and the FS separation capillaries protruded 10 and 8 mm from the electrodes, respectively. To achieve exactly these lengths, the capillary ends extending from the plastic CE cartridge were measured and were 52 mm for the inlet end and 50 mm for the outlet end (see the photograph in ref[3]). FS capillaries for sample vial filling with DBS elution solvents were also from Polymicro Technologies, had i.d.s of 50, 75, 100, 150, and 200 μm (375 μm o.d.) and L_{tot} was 30 or 50 cm. They were placed in another cartridge and the capillary ends extended from the CE cartridge by 50 and 53.5 mm at the inlet and the outlet side, respectively.

Precise liquid handling was achieved by an additional removal of 2 mm of polyimide coating on both capillary ends (in both cartridges). All capillary flushing, conditioning, and filling procedures were carried out at 950 mbar (the maximum pressure of the Agilent 7100 CE).

RESULTS AND DISCUSION

Sample Vial, DBS Size, and Eluent Volume

Due to the minimally invasive and simple blood sampling, DBSs have been shown highly attractive for the patient-centric blood collection recently.[4-5] The transport chain of remotely collected clinical samples assumes their delivery to a laboratory by mail, thus, non-fragile and easily transportable PP sample vials[6] were applied in our actual experiments. The sample vials are compatible with the Agilent 7100 CE autosampler and are suited for processing minute volumes of dried blood samples with minute volumes of elution solvents. In the actual experiments, DBSs were formed by collecting a mere 5 µL of capillary blood and were processed by eluting the spots with 100 µL of elution solvents, which enabled sufficient eluate volumes for replicate CE injections.[6] The final dilution factor was 20 and was suitable for the determination of therapeutic concentrations of NSAIDs as well as endogenous concentrations of amino acids in DBSs.

DBS Elution Solvent

The elution efficiency of target analytes as well as the complexity of the resulting DBS eluate depend strongly on the composition of the elution solvent because specific blood components elute differently into different solvents. For example, DI water efficiently dissolves DBS matrices, thus aqueous DBS eluates contain high concentrations of small ions, lipids, proteins, and cellular and other macromolecular compounds next to the low concentrations of target analytes. The presence of the blood matrix in the DBS eluates can be detrimental to most analytical techniques, including CE. Indeed, aqueous DBS eluates were not suitable for counter-electroosmotic CE of anionic analytes previously.[3] On the other hand, organic solvents precipitate blood macromolecular components, which are not released into organic DBS

eluates. Such eluates are then well suited for the direct analysis by separation techniques, however, organic solvents might suppress elutions of hydrophilic analytes.

Various elution solvents were examined in this study and corresponding results are presented in the main manuscript.

Flow-Through Characteristics of Various FS Capillaries for Autonomous DBS Elutions

A comprehensive evaluation of the flow rates through filling capillaries with various lengths and i.d.s is presented in Figure S1 and S2. The resulting curves offer a fundamental knowledge about the liquid handling by an unmodified Agilent 7100 CE system and might be helpful for tailored elutions of DBSs and other dried material spots (DMSs). The requirements on the number of DBSs/DMSs in the CE carousel, collected volumes of biological samples, eluate volumes, dilution factors, and complexity of the resulting eluates might differ significantly for various analytical methods. Each method might, thus, require a specific volume of the elution solvent (ranging from tens to thousands µL) and benefit from the high flexibility of the autonomous liquid handling using capillaries with different dimensions. In addition, various collection devices[7-10] and various dried biological samples may require specific elution times, and the capillaries inappropriate for the actual study (i.e. with too low or too high i.d., or capillaries with even more extreme dimensions) might be optimal for other applications.

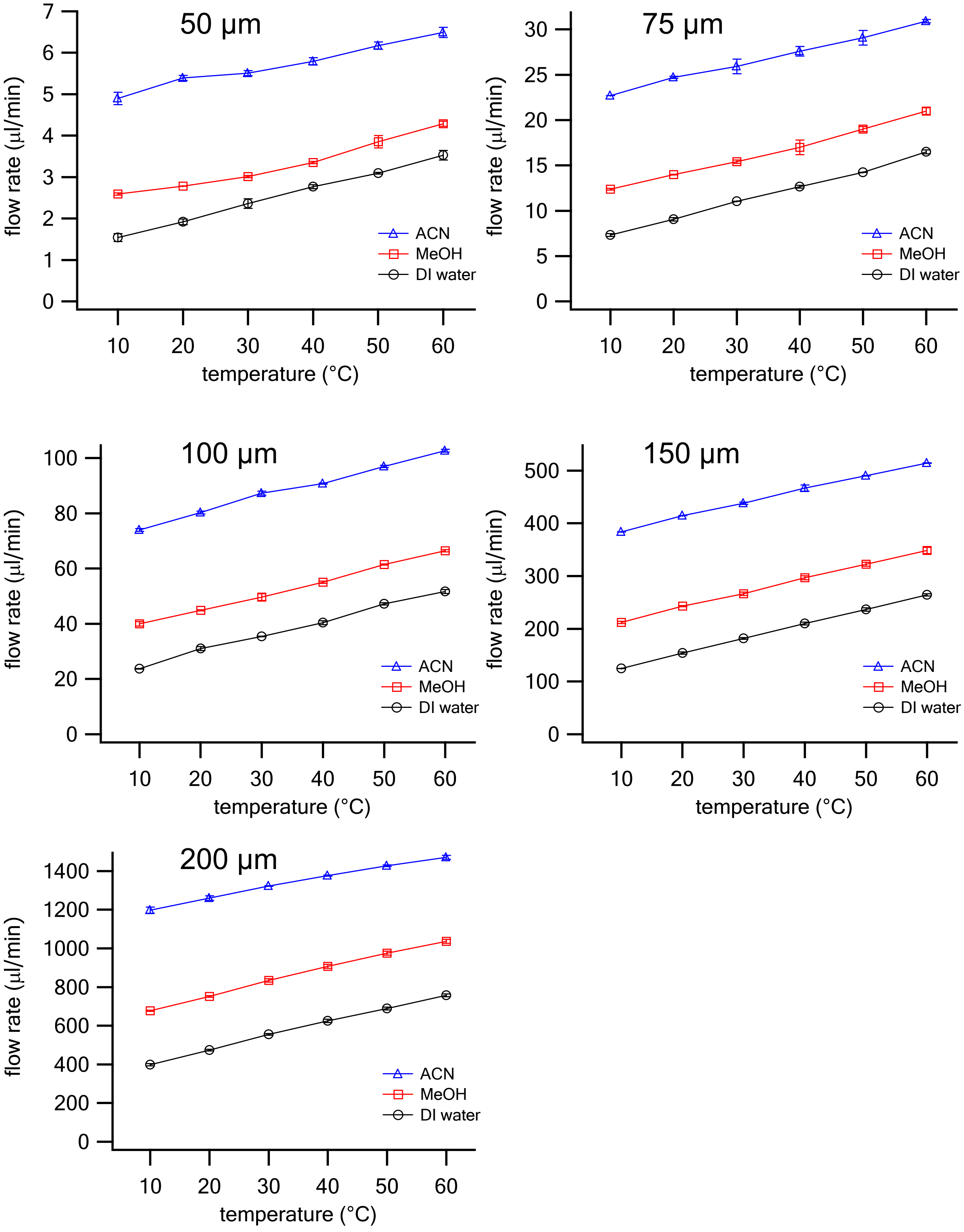

Figure S1. The effect of the capillary i.d. on the flow rate of DI water, MeOH and ACN through 50 cm long FS capillaries. Pressure, 950 mbar was generated by the Agilent 7100 CE system and applied to the inlet CE vial, $n = 3$.

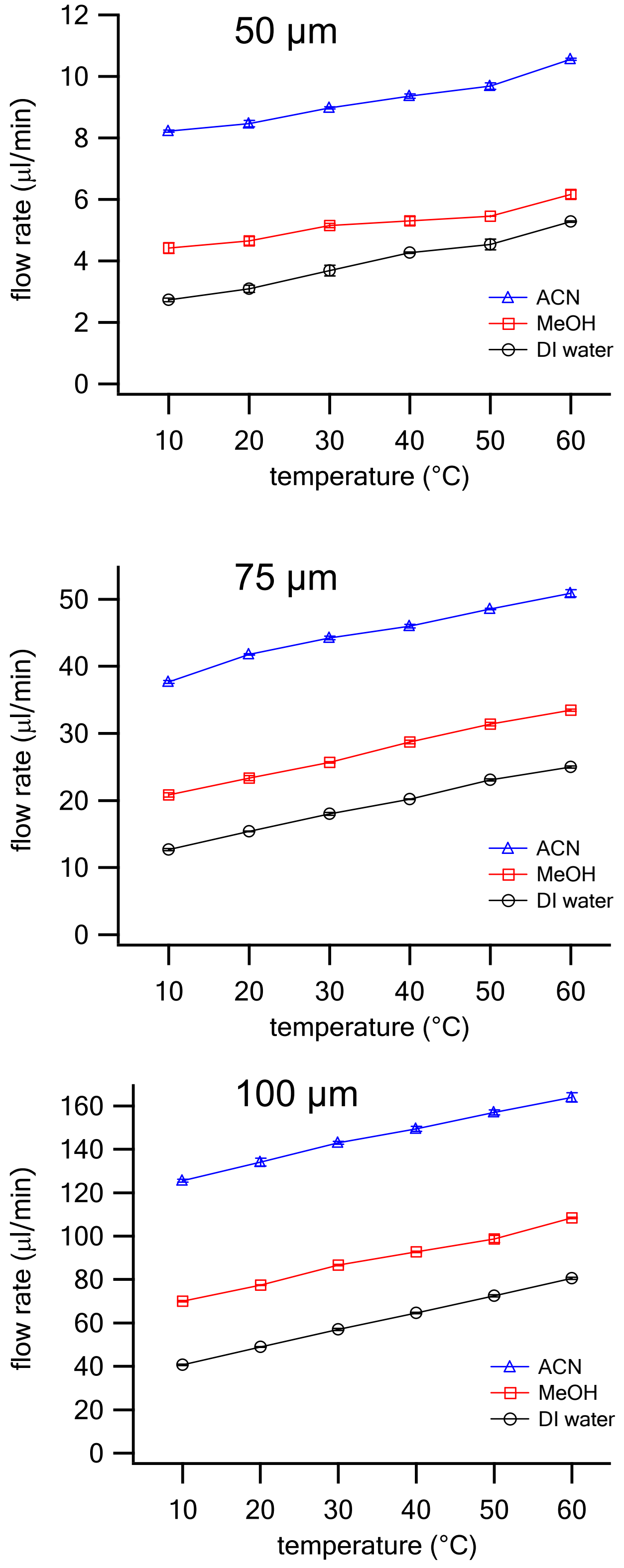

Figure S2. The effect of the capillary i.d. on the flow rate of DI water, MeOH and ACN through 30 cm long FS capillaries. Pressure is the same as in Figure S1, $n = 3$.

BGE Solution Optimization for CE-UV Determination of NSAIDs

The following three variables (concentration of acetic acid, sodium acetate, and ACN in the BGE solution) were optimized to achieve a baseline separation of two unknown matrix components (labeled by asterisks in Figure S3) from ibuprofen and diclofenac. Other criteria were the separation time and electric current, which were kept at a minimum to enable high-throughput DBS analyses and to minimize Joule heating, respectively. The previous BGE solution,[2] i.e. 30 mM acetic acid, 30 mM sodium acetate and 30% (v/v) ACN did not enable full separation of matrix components and the four NSAIDs, and the BGE solution composition was, thus, further optimized. By increasing the acetic acid concentration (to e.g. 60 mM), the separations were faster, while they were slower for higher sodium acetate concentration (e.g. 60 mM). The electric current increased significantly for higher sodium acetate concentrations while acetic acid concentration had only a negligible effect on the current. The migration of the matrix components was considerably affected by the content of ACN, moving their positions forward and backward (in relation to the analytes) for lower (20% v/v) and higher (40% v/v) ACN concentrations, respectively. Baseline separation of all analytes and matrix peaks was achieved in the BGE solution consisting of 30 mM sodium acetate, 60 mM acetic acid and 20% (v/v) ACN. The BGE solution apparent pH was 4.8 and this BGE solution was used for all subsequent CE-UV measurements. The separation time was approx. 4 min and the electric current was approx. 60 μA.

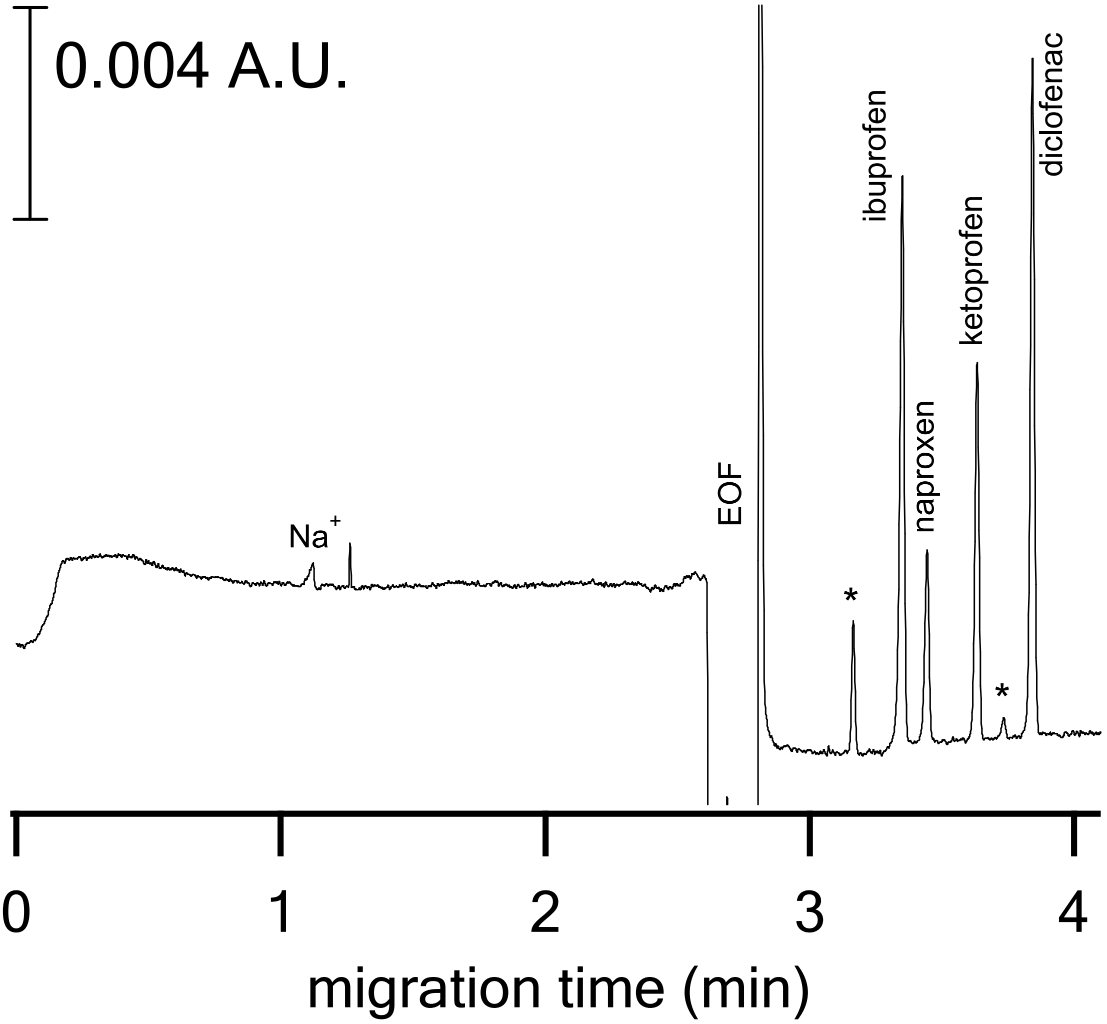

Figure S3. Electropherogram for the CE-UV determination of NSAIDs in a DBS. CE conditions: FS capillary, 75 µm i.d., L_{tot} = 50 cm, L_{eff} = 41 cm; BGE solution, 30 mM sodium acetate, 60 mM acetic acid, and 20% (v/v) ACN, apparent pH 4.8; separation voltage, + 27.5 kV; cartridge temperature, 30 °C; injection, 50 mbar for 7 s; detection wavelength, 200 nm. DBS conditions: 5 µL DBS spiked with 25 mg/L of NSAIDs and eluted with 80 µL of ACN and 20 µL of DI water (added consecutively) at 1200 rpm for 20 min. EOF – electroosmotic flow, * – unknown matrix compounds.

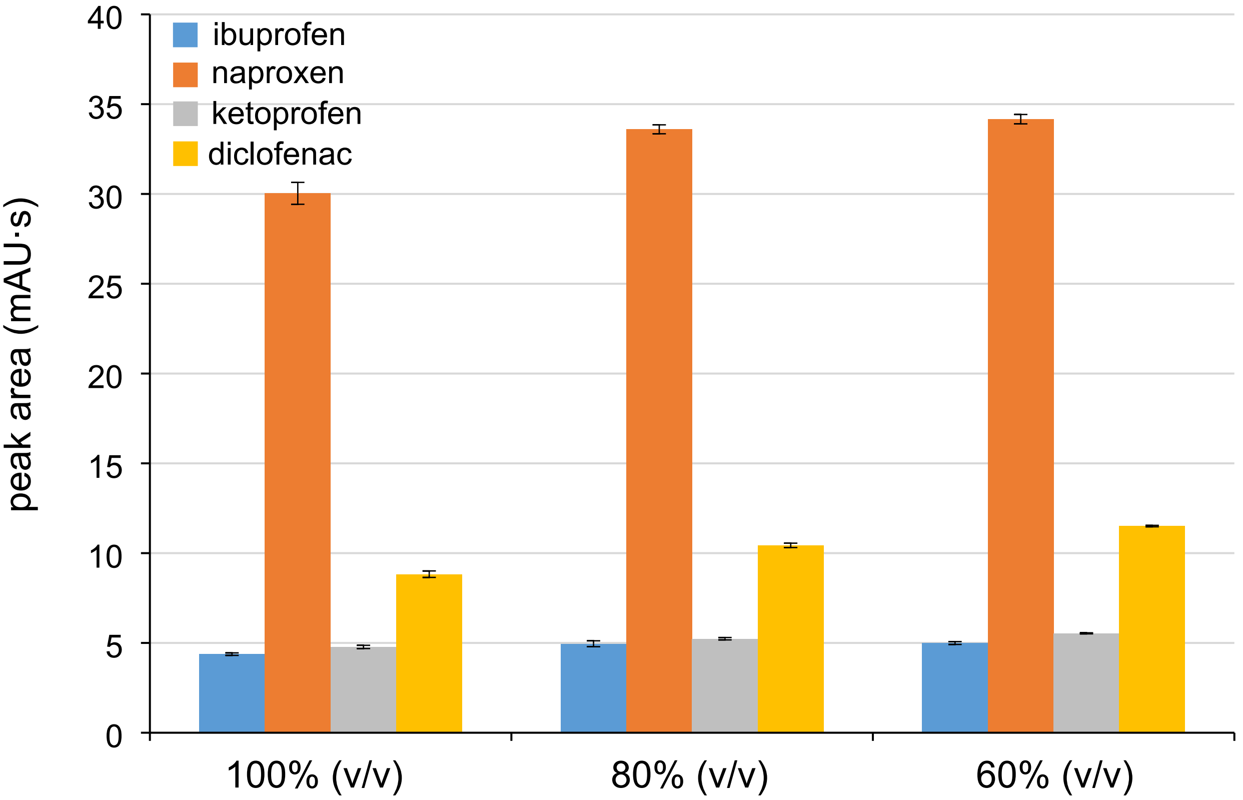

Figure S4. The effect of the MeOH content (v/v) in the elution solvent on the NSAIDs elution from DBSs. DBS conditions: 5 µL DBS eluted in 100 µL of various elution solvents (organic solvent and DI water added consecutively) by agitation at 1200 rpm for 20 min, $n = 3$.

Evaporation of Elution Solvents

The low volumes of the eluate solutions in the sample vials, especially organic solutions, might be prone to evaporation and the stability of their volumes was examined for DI water, 80% (v/v) ACN, and 60% (v/v) MeOH ($n = 3$). New PP vials and new PEO caps were used for the experiments. The elution solvents were transferred by CE to the vials (PEO caps were pierced two-times by the prepuncher) and their volumes were determined immediately after the transfer and after standing for 5 h in the autosampler carousel, which was operated in a standard CE separation mode. The volumes decreased only negligibly for DI water (0.3 ± 0.1%), but they decreased considerably for ACN and MeOH solutions (5.7 ± 1.9% and 4.3 ± 1.0%). On the contrary, DI water, ACN, and MeOH volumes did not change in vials closed with unpierced

PEO caps and suggested evaporation of the organic solvents through the holes made by the CE prepuncher. To eliminate the evaporation, a thin septum (1 × 10 mm) was inserted into the cap, which resealed the vial after each opening by the prepuncher. Volumes of all elution solvents decreased by less than 0.4 ± 0.1% (for this arrangement and 5 h CE operation time) and using new PP vials and new PEO caps with septum is thus always recommended. If necessary, a further improvement in volume stability might be achieved by the application of an external cooling of the autosampler carousel.

Maximum Sample Capacity of the CE Carousel

The CE carousel of the Agilent 7100 CE controls up to 50 vial positions. Two positions are left unused by the instrument for the CE system (typically position #49 and #50) and the remaining 48 positions can be filled with CE solutions and DBS sample vials. By using two different cartridges (one for DBS elutions and the other for DBS eluate analyses) the positions in the carousel used for the DBS elution solvents in the first sequence (positions #2 through #6) can be used for CE operational solutions in the second sequence. The number of positions for DBS samples can thus be maximized and in the actual set-up, a maximum of 42 DBS samples (positions #7 through #48) could be processed and analyzed with the tailored sequences. The overview of the carousel positions and their occupation in the two sequences (DBS elution and DBS analysis) for the maximum sample throughput is reported in Table S1. In our experiments, 36 DBS samples were processed and analyzed using two previously optimized sequences, and the distribution of the solutions in the CE carousel is presented in Tables S2 and S4.

Table S1. Solutions and their positions in the CE carousel during DBS elution and DBS analysis for the maximum sample throughput (up to 42 DBS samples).

Position	DBS elution	DBS analysis
1	Waste	Waste
2	Air	100 mM NaOH
3	DBS elution solvent	BGE solution (CE separation)
4	DBS elution solvent	BGE solution (CE separation)
5	DBS elution solvent	BGE solution (inject)
6	DBS elution solvent	BGE solution (capillary flush)
7 – 48	DBS sample	DBS sample
49	CE system – unused	CE system – unused
50	CE system – unused	CE system – unused

Table S2. Solutions, their volumes, and their positions in the CE carousel for the automated CE-UV determination of NSAIDs in 36 DBS samples.

Solution	Position	Volume (μL)	Vial material
Waste (prefilled with DI water)	1	400	Glass
100 mM NaOH	2	700	Glass
Air	4	Empty vial	PP
BGE solution (CE separation)	5	1400	Glass
BGE solution (CE separation)	6	1400	Glass
BGE solution (inject)	7	1400	Glass
BGE solution (capillary flush)	8	750	Glass
ACN (DBS elution solvent)	9	1500	Glass
ACN (DBS elution solvent)	10	1500	Glass
ACN (DBS elution solvent)	11	1500	Glass
DI water (DBS elution solvent)	12	1500	Glass
DBS sample	13 – 48	Vial with a DBS	PP

Ibuprofen Pharmacokinetic Curve

A high time resolution of collected blood samples is crucial for TDM. The presented concept might be attractive for TDM due to the minimum requirements on blood volumes, suitability for blood collection at a high frequency, and subsequent rapid and automated analysis. DBS samples were collected at time 0 min (i.e. just before the oral administration of Ibalgin tablet (400 mg ibuprofen)), then every 15 min (until 120 min), and then at 160, 220, 280, and 440 min after the administration. Because the collected blood volume was only 5 µL, three DBSs were formed for each collection time from a single finger prick. The blank DBSs (0 min) were drug-free and confirmed the presence of no ibuprofen in the blood. The 36 DBSs collected after Ibalgin administration were processed and analyzed using the previously optimized automated sequence and the resulting pharmacokinetic curve is depicted in Figure S5. The curve showed a rapid increase of blood ibuprofen between 30 and 60 min, maximum ibuprofen concentration at 75 min, and a gradual decrease of ibuprofen concentrations until about 8 h after the drug administration. Representative electropherograms of DBSs collected at 0, 45, 75, and 160 min after Ibalgin administration are depicted in Figure S6. The curve profile was similar to pharmacokinetic curves determined for ibuprofen in blood plasma,[11] nevertheless, ibuprofen concentrations in DBSs were 2 – 3-times lower. The differences in absolute ibuprofen concentrations in DBS vs. blood plasma were caused by the different distribution of the drug among erythrocytes and plasma (ibuprofen content is lower in erythrocytes) and results in lower whole blood ibuprofen concentrations.[12]

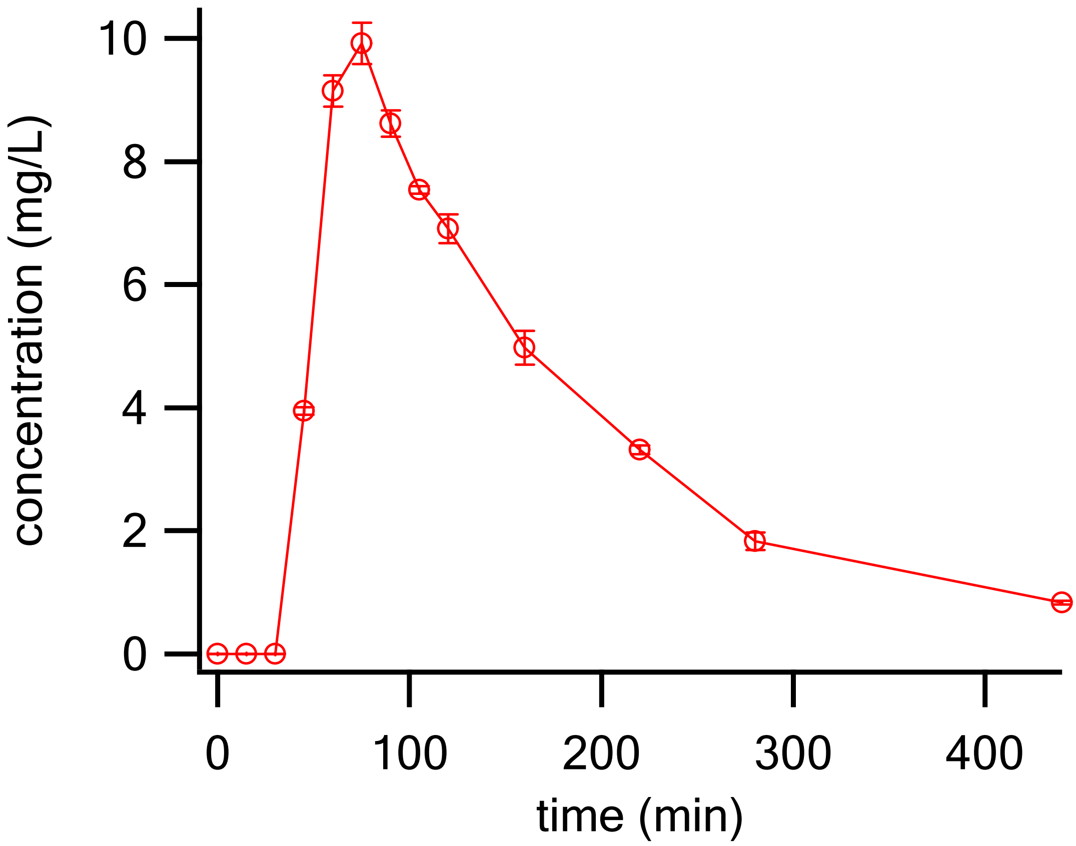

Figure S5. A pharmacokinetic curve for the autonomous determination of ibuprofen in DBSs after oral administration of Ibalgin tablet. DBS conditions: 5 μL DBS eluted in ~ 81 μL of ACN and ~ 21 μL of DI water (added consecutively) by the CE in-vial elution procedure, *n = 3*.

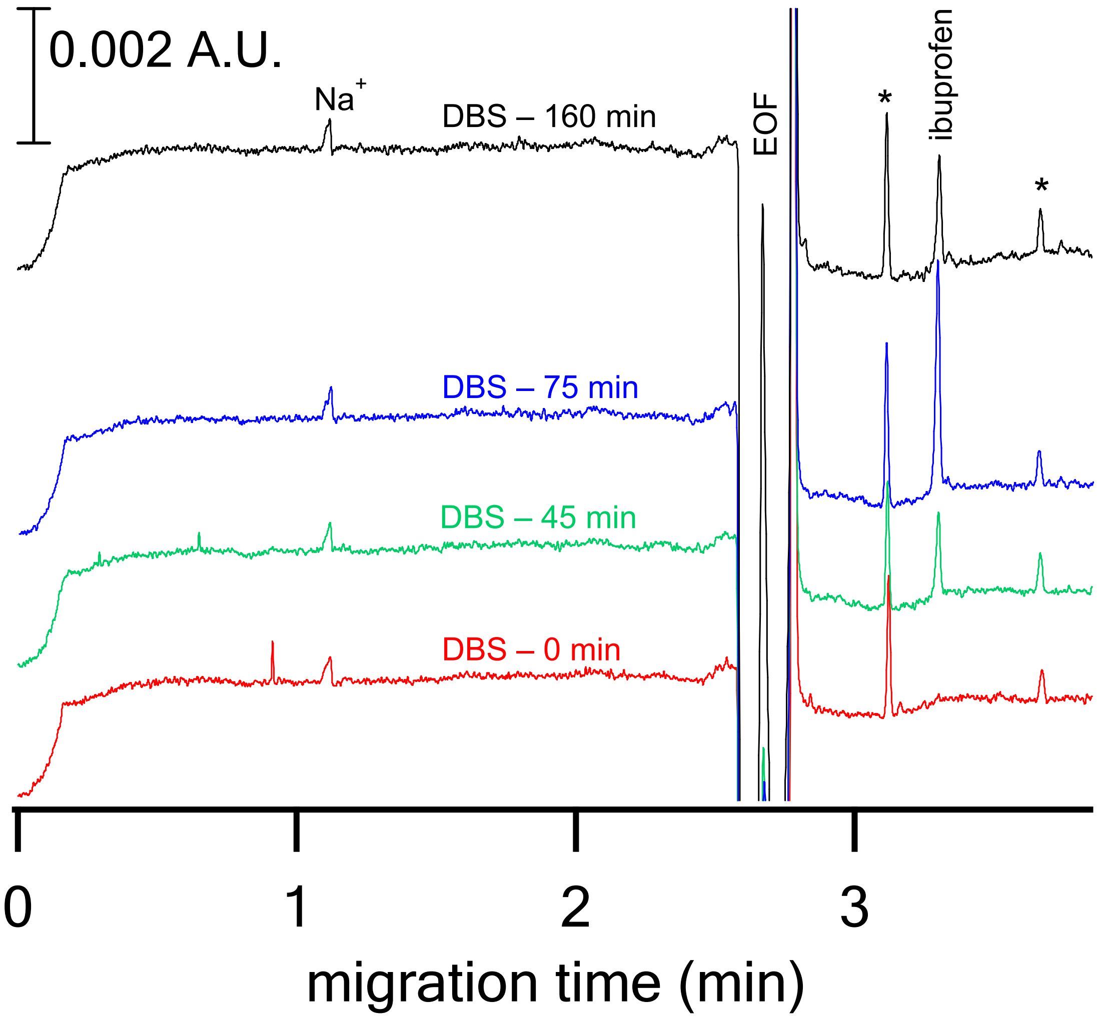

Figure S6. Electropherograms for the CE-UV determination of ibuprofen in DBSs collected at different times after oral administration of Ibalgin tablet. CE conditions and DBS conditions are the same as in Figure S3 and S5, respectively. EOF – electroosmotic flow, * – unknown matrix compounds.

Table S3. Analytical parameters of the automated CE-UV determination of NSAIDs in DBS samples.

Analyte	Concentration[a] (mg/L)	RSD PA (%)	RSD t_m (%)	Δ_{conc} (%)	R^2	LOD[a] (mg/L)	LOQ[a] (mg/L)
IBU	10	2.7[b]	0.4[b]		0.999	0.4	1.32
	10	3.5[c]	0.8[c]				
	endogenous	3.6[d]	0.6[d]	–4.7[e]			
	1.25	4.9[f]	0.5[f]				
	5	3.4[f]	0.4[f]				
	25	3.4[f]	0.5[f]				
	50	2.2[f]	0.2[f]				
NAP	10	2.5[b]	0.5[b]		0.999	0.16	0.52
	10	2.4[c]	0.8[c]				
	1.25	3.7[f]	0.5[f]				
	5	1.1[f]	0.4[f]				
	25	2.2[f]	0.5[f]				
	50	2.0[f]	0.2[f]				
KET	10	1.6[b]	0.5[b]		0.998	0.6	2.0
	10	4.1[c]	0.8[c]				
	1.25	5.1[f]	0.5[f]				
	5	3.8[f]	0.4[f]				
	25	2.1[f]	0.5[f]				
	50	2.3[f]	0.2[f]				
DIC	10	1.7[b]	0.5[b]		0.998	0.4	1.32
	10	3.8[c]	0.9[c]				
	1.25	4.2[f]	0.5[f]				
	5	2.0[f]	0.4[f]				
	25	2.6[f]	0.5[f]				
	50	2.4[f]	0.3[f]				

[a] calculated for undiluted capillary blood
[b] one DBS eluate analyzed ten times (*n = 10*)
[c] three DBSs analyzed on three different days (*n = 9*)
[d] four different DBS eluates analyzed three times each (*n = 12*)
[e] concentration difference between a 28-days old and a fresh DBS
[f] three different DBS eluates analyzed three times each (*n = 9*)
PA – peak area; t_m – migration time; IBU – ibuprofen, NAP – naproxen, KET – ketoprofen; DIC – diclofenac

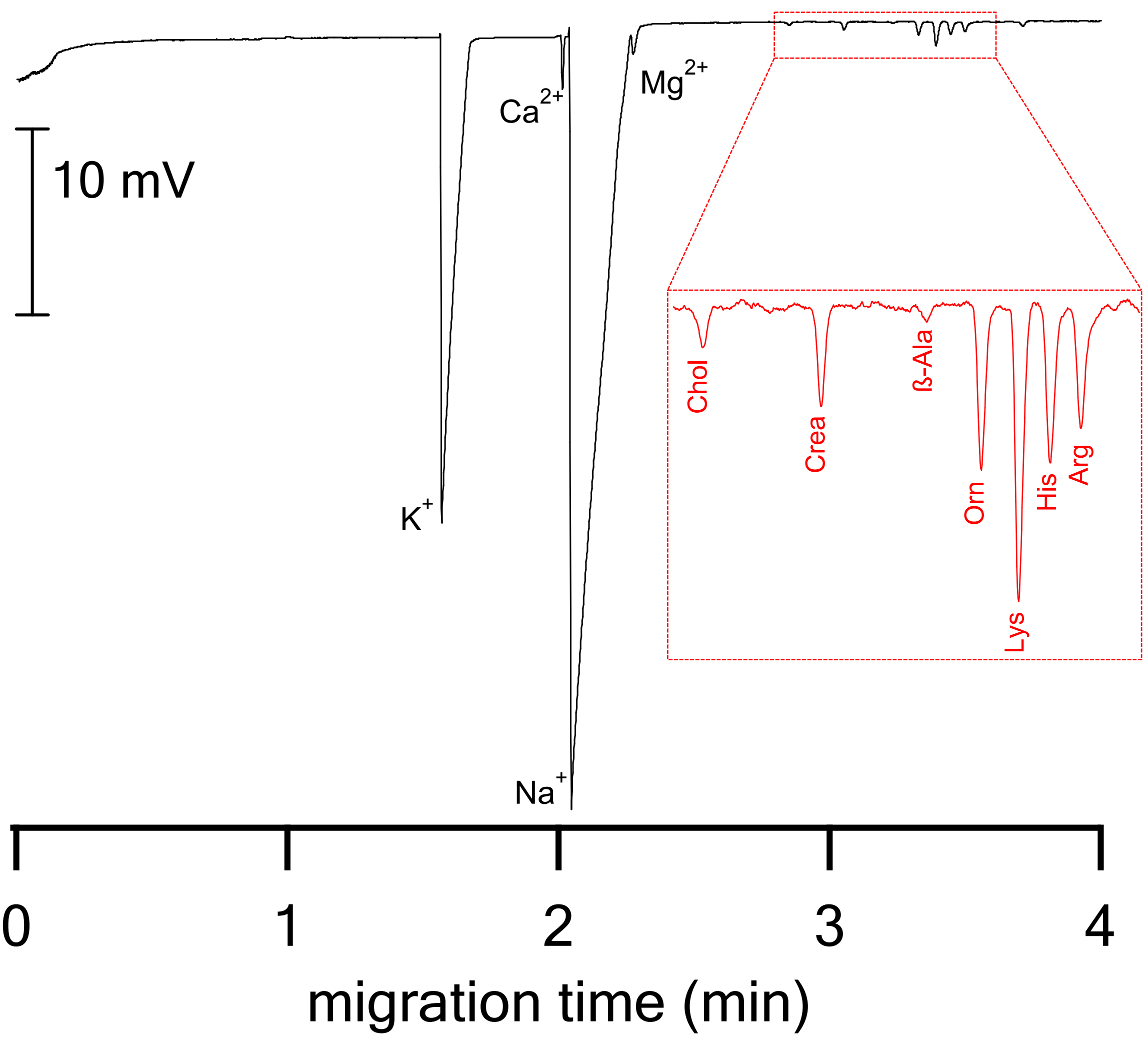

Figure S7. Electropherogram for the CE-C[4]D determination of inorganic cations and amino acids in a DBS. CE conditions: FS capillary, 25 µm i.d., L_{tot} = 50 cm, L_{eff} = 35 cm; BGE solution, 0.4 M acetic acid and 0.1% (v/v) Tween 20, pH 2.6; separation voltage, + 25 kV; cartridge temperature, 30 °C; injection, 100 mbar for 10 s. DBS conditions: 5 µL DBS eluted with 60 µL of MeOH and 40 µL of 20-fold diluted BGE (added consecutively) at 1200 rpm for 60 min.

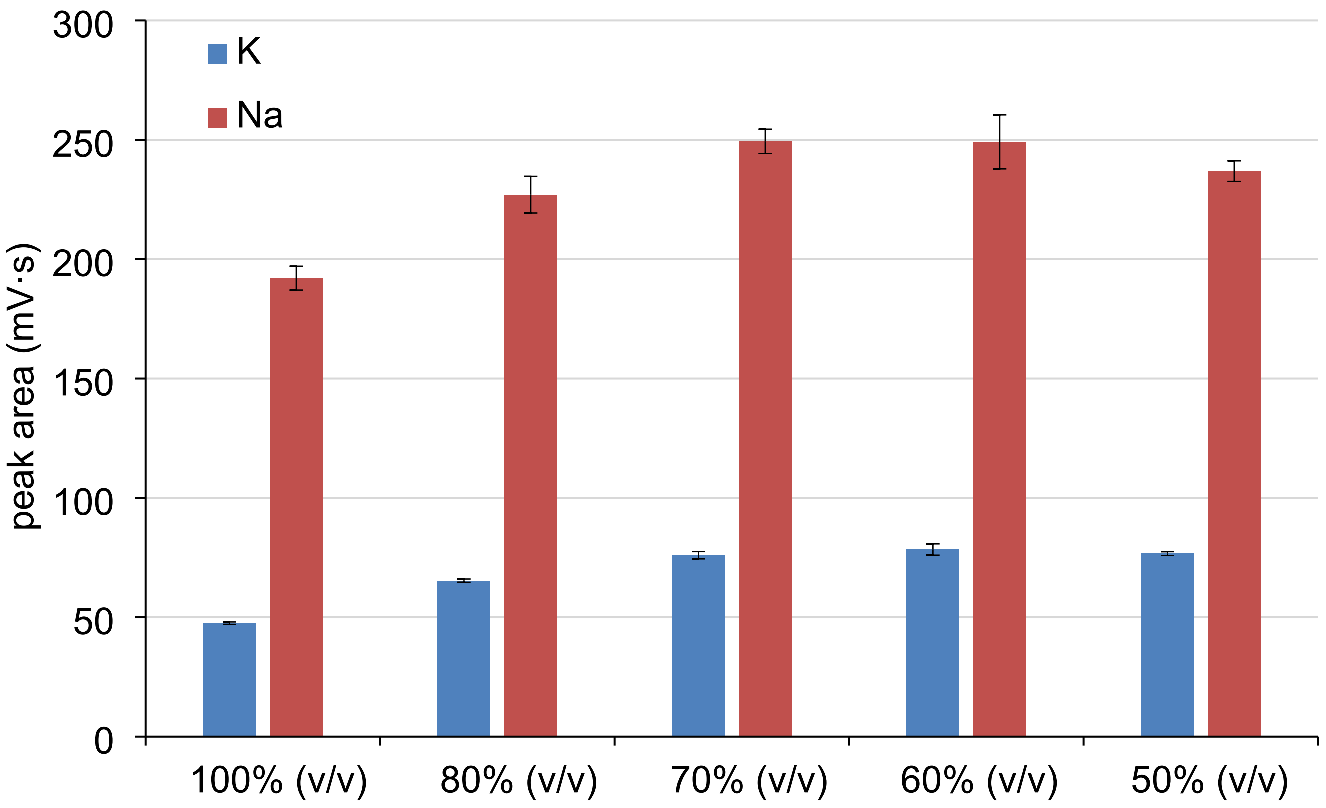

Figure S8. The effect of the MeOH content in the elution solvent on the elution of K^+ and Na^+ from DBSs. DBS conditions: 5 µL DBS eluted in 100 µL of various MeOH elution solvents (MeOH and DI water added consecutively) by agitation at 1200 rpm for 60 min, $n = 3$.

Table S4. Solutions, their volumes, and their positions in the CE carousel for the automated CE-C4D determination of amino acids in 36 DBS samples.

Solution	Position	Volume (µL)	Vial material
Waste (prefilled with DI water)	1	400	Glass
100 mM NaOH	2	700	Glass
Air	4	Empty vial	PP
BGE solution (CE separation)	5	1400	Glass
BGE solution (CE separation)	6	1400	Glass
BGE solution (inject)	7	1400	Glass
BGE solution (capillary flush)	8	700	Glass
MeOH (DBS elution solvent)	9	1500	Glass
MeOH (DBS elution solvent)	10	1500	Glass
Diluted BGE (DBS elution solvent)	11	1500	Glass
Diluted BGE (DBS elution solvent)	12	1500	Glass
DBS sample	13 – 48	Vial with a DBS	PP

Table S5. Analytical parameters of the automated CE-C^{4}D determination of rapid amino acids in DBS samples.

Analyte	Concentration[a] (μM)	RSD PA (%)	RSD t_m (%)	Δ_{conc} (%)	R^2	LOD[a] (μM)	LOQ[a] (μM)
Chol	endogenous	8.5[b]	0.6[b]	–4.3[c]	0.9998	4	13.3
	endogenous	6.7[d]	1.5[d]				
	20	1.1[e]	0.5[e]				
	100	3.0[e]	0.5[e]				
	400	0.5[e]	0.7[e]				
Crea	endogenous	5.0[b]	0.7[b]	+8.5[c]	0.9992	5	16.7
	endogenous	3.3[d]	1.6[d]				
	20	3.1[e]	0.5[e]				
	100	3.9[e]	0.5[e]				
	400	0.3[e]	0.7[e]				
ß-Ala	endogenous	19.2[b]	0.8[b]	+8.4[c]	0.9992	6	20.0
	endogenous	12.9[d]	1.8[d]				
	20	12.3[e]	0.5[e]				
	100	3.0[e]	0.5[e]				
	400	0.5[e]	0.7[e]				
Orn	endogenous	6.7[b]	0.8[b]	–10.9[c]	0.9960	5	16.7
	endogenous	9.3[d]	1.9[d]				
	20	2.0[e]	0.5[e]				
	100	4.5[e]	0.5[e]				
	400	1.9[e]	0.7[e]				
Lys	endogenous	6.9[b]	0.8[b]	–1.1[c]	0.9992	5	16.7
	endogenous	6.2[d]	1.9[d]				
	20	2.1[e]	0.5[e]				
	100	3.6[e]	0.5[e]				
	400	0.8[e]	0.7[e]				
Arg	endogenous	8.7[b]	0.8[b]	–33.7[c]	0.9998	5	16.7
	endogenous	6.6[d]	2.0[d]				
	20	1.2[e]	0.6[e]				
	100	3.4[e]	0.6[e]				
	400	0.5[e]	0.7[e]				
His	endogenous	7.5[b]	0.9[b]	+9.9[c]	0.9989	5	16.7
	endogenous	12.3[d]	2.0[d]				
	20	2.6[e]	0.6[e]				
	100	6.5[e]	0.6[e]				
	400	2.3[e]	0.7[e]				

[a] calculated for undiluted capillary blood
[b] eighteen DBS eluates (*n = 18*)
[c] concentration difference between a 28-days-old and a fresh DBS
[d] three DBSs analyzed on three different days (*n = 9*)
[e] three DBS eluates analyzed three times each (*n = 9*)
PA – peak area; t_m – migration time

REFERENCES

1. Dvořák, M.; Ryšavá, L.; Kubáň, P., Capillary Electrophoresis with Capacitively Coupled Contactless Conductivity Detection for Quantitative Analysis of Dried Blood Spots with Unknown Blood Volume. *Anal. Chem.* **2020,** *92* (1), 1557-1564.

2. Abu-Rabie, P.; Denniff, P.; Spooner, N.; Chowdhry, B. Z.; Pullen, F. S., Investigation of Different Approaches to Incorporating Internal Standard in DBS Quantitative Bioanalytical Workflows and Their Effect on Nullifying Hematocrit-Based Assay Bias. *Anal. Chem.* **2015,** *87* (9), 4996-5003.

3. Ryšavá, L.; Dvořák, M.; Kubáň, P., Dried Blood Spot Self-Sampling with Automated Capillary Electrophoresis Processing for Clinical Analysis. *Angew. Chem. Int. Ed.* **2021,** *60* (11), 6068-6075.

4. Protti, M.; Mandrioli, R.; Mercolini, L., Quantitative microsampling for bioanalytical applications related to the SARS-CoV-2 pandemic: Usefulness, benefits and pitfalls. *J. Pharm. Biomed. Anal.* **2020,** *191*, 113597.

5. Rudge, J.; Kushon, S., Volumetric absorptive microsampling: its use in COVID-19 research and testing. *Bioanalysis* **2021,** *13* (24), 0102.

6. Moravčík, O.; Dvořák, M.; Kubáň, P., Autonomous capillary electrophoresis processing and analysis of dried blood spots for high-throughput determination of uric acid. *Anal. Chim. Acta* **2023,** *1267*, 341390.

7. Denniff, P.; Spooner, N., Volumetric Absorptive Microsampling: A Dried Sample Collection Technique for Quantitative Bioanalysis. *Anal. Chem.* **2014,** *86* (16), 8489-8495.

8. Leuthold, L. A.; Heudi, O.; Deglon, J.; Raccuglia, M.; Augsburger, M.; Picard, F.; Kretz, O.; Thomas, A., New Microfluidic-Based Sampling Procedure for Overcoming the Hematocrit Problem Associated with Dried Blood Spot Analysis. *Anal. Chem.* **2015,** *87* (4), 2068-2071.

9. Neto, R.; Gooley, A.; Breadmore, M. C.; Hilder, E. F.; Lapierre, F., Precise, accurate and user-independent blood collection system for dried blood spot sample preparation. *Anal. Bioanal. Chem.* **2018,** *410* (14), 3315-3323.

10. Velghe, S.; Stove, C. P., Evaluation of the Capitainer-B Microfluidic Device as a New Hematocrit-Independent Alternative for Dried Blood Spot Collection. *Anal. Chem.* **2018,** *90* (21), 12893-12899.

11. Moghadamnia, Y.; Kazemi, S.; Rezaee, B.; Rafati-Rahinizadeh, M.; Ebrahimpour, S.; Aghapour, F., New formulation of ibuprofen on absorption-rate: A comparative bioavailability study in healthy volunteers. *Casp. J. Int. Med.* **2019,** *10* (2), 150-155.

12. Sochor, J.; Klimeš, J.; Sedláček, J.; Zahradníček, M., Determination of ibuprofen in erythrocytes and plasma by high-performance liquid-chromatography. *J. Pharm. Biomed. Anal.* **1995,** *13* (7), 899-903.
